# Editorial: Pediatric thoracic surgery

**DOI:** 10.3389/fped.2023.1132803

**Published:** 2023-01-18

**Authors:** Mario Lima

**Affiliations:** Pediatric Surgery, IRCCS Sant'Orsola Hospital University of Bologna, Bologna, Italy

**Keywords:** pediatric, thoracic, surgery, mini-invasive, congenital, airway malformations, esophageal atresia, ewing sarcomas

Editorial on the Research Topic Pediatric thoracic surgery

In the last decades pediatric thoracic surgery significantly developed, extending minimally invasive approaches such as thoracoscopy to infancy and childhood. This progress is mainly due to the introduction of specific devices for the management of little spaces and little anatomical structures. Pediatric thoracoscopic surgery has increasingly become important in clinical practice and now it represents a well-established approach for infants and children and it is considered, by most thoracic surgeons, as the best choice for many procedures. Pediatric thoracoscopic surgery allows to reduce pain and morbidity and to avoid the long-term consequences of a thoracotomy in an infant or a small child.

The introduction of Robot-assistance in pediatric thoracoscopic surgery has represented the latest instrumental innovation. Thus, innovations in minimally invasive surgery still need to be reported and validated. Furthermore, the development of this new minimally invasive approach requires a change in the modalities of training in pediatric thoracic surgery.

Nevertheless, there still are many unsolved questions on many conditions, like anesthetic approach during thoracoscopic surgery or management of congenital disease as cystic pulmonary malformations (CPM) or esophageal atresia (EA).

Thoracoscopy represents the most challenging area of pediatric minimally invasive surgery and a standardized training program would be advisable. Macchini et al. proposed a standardized training program is highly desirable to learn how to safely perform advanced pediatric thoracoscopy. It is based on a 2 year four-step program that consisted in: (1) theoretical part; (2) experimental training; (3) training in centers of reference; (4) personal operative experience.

Esophageal atresia (EA), although a rare congenital anomaly, represents one of the most common gastrointestinal birth defects. Due to its complexity, the scientific debate on the proper management of this condition and its consequences is still open. Recent developments in neonatal intensive care have significantly increased survival rates over the last decade also in premature babies and in those with associated malformations. Based on that Evanovich et al. proposed a need for a proper risk stratification in this unique population. In this study the authors addressed EA types, disease severity stratification (according to American Society of Anesthesiologists (ASA) and Pediatric Risk Assessment (PRAm) scores), and mortality in a retrospective cohort at a single institution. Despite a wider PRAm score distribution in infants born with EA, ASA scores remain the gold standard in assessing underlying disease severity stratification.

Thoracoscopic approach has been presented by Yong et al. as a valuable approach in managing esophageal diverticula after EA with tracheo-esophageal fistula. Esophageal diverticulum is an extremely rare complication of EA and is a clear indication for diverticulectomy. In their series of 4 cases of esophageal diverticula thoracoscopy has been demonstrated to be a safe and effective approach for diverticulectomy.

In another article Ladefoged et al. aim to demonstrate whether the potential benefits of prophylactic intraoperative chest tube (IOCT) overweigh the potential harms. They conducted a systematic review and a meta-analysis on the most common research platform. Randomized clinical trials assessing the effect of a prophylactic IOCT during primary surgical repair of EA and observational studies were included. In their research the authors concluded that there is no evidence of a beneficial effect of placing a prophylactic IOCT during primary surgical repair of EA.

EA is strictly connected to different degrees of tracheomalacia (TM) in up to 87% of cases. Van Tuyll van Serooskerken et al. reported their own experience on performing primary posterior tracheopexy in EA in moderate and severe cases of ™ detected at first pre-operative bronchoscopy. The procedure has been performed in 36 cases and it impacted positively on the outcome of these patients by decreasing the rate of respiratory tract infections.

Moving forward to another congenital condition as pulmonary airway malformations we could appreciate the contribution given by Koga et al. In their research they would like to describe the use of an additional trocar (AT) in the lower thorax during thoracoscopic pulmonary lobectomy. Comparing two different populations based on the use or not of an AT they concluded that an AT and switching facilitated posterior dissection during TPL in children with congenital pulmonary airway malformation enhancing safety and efficiency.

Another crucial point in the management of congenital pulmonary airway malformations is whether to operate on asymptomatic patients. To answer this question Liu et al. proposed a novel point of view focusing on the proportion of hidden infection in congenital pulmonary airway malformations and its effect on surgery. In their study, patients with hidden infection accounted for 32% of all asymptomatic congenital pulmonary airway malformations patients. Hidden infection would increase the difficulty and risk of surgery and cause more surgical complications.

Oncology is one of the most promising frontier for pediatric surgeons who would like to extend the advantages of minimally invasive approaches also to those patients. Riccipetitoni et al. in their article give us a very interesting overview on their experience on application of thoracoscopy in pediatric malignancy. After reviewing 38 patients they concluded that thoracoscopy represents a valuable tool for diagnostic and therapeutic procedures in pediatric oncology that should be performed by expert surgeons. They also suggest that the advent of robotic surgery represents a new challenge that may further implement the advantages of the thoracoscopic approach.

Talking about tumors the key point of the treatment is the rate of survival. Ewing sarcomas of the chest wall represent an highly aggressive pediatric malignancies. Basharkhah et al. in their study reported a very promising results in terms of survival after an innovative multi-modal treatment. They sustain as specific oncological (neo)adjuvant treatment and multi-disciplinary surgery performing radical en-bloc resections and simultaneous chest wall repair contribute to a long-time survival of children and adolescents with Ewing sarcoma of the chest wall up to 89%. The main limit of the study is the small number of cases but the promising results should encourage many centers to this innovative multi-modal management of this tumor.

Last point of this research topic collection on thoracic surgery is congenital chest wall malformations. Nuss procedure is still considered a very challenging procedure to correct pectus excavatum. In their contribution McCoy and Hollinger focused on the anesthesiological point of view in the managment of these patients during operation. In particular they advocate as the use cryonalagesia, instead of epidural, and lung Isolation with the EZ-Blocker™, could improve surgical and clinical outcome of these patients.

The latest contribution is the one of Beigee at al. In this case report the authors present the first case of chest wall reconstruction by utilizing cryopreserved sternum in children. A 5-year old girl affected by hemangioma, receveid resection of the sternum; but the large anterior chest wall defect was reconstructed a by a cryopreserved sternal allograft. In the follow-up of the patient, there was no instability of the chest wall and acceptable cosmetic results.

## Author contributions

ML has made a substantial contribution to the concept or design of the article; ML Drafted the article and revised it critically for important intellectual content, and approved the version to be published. All authors contributed to the article and approved the submitted version.

